# Robert C. Turner

**Published:** 1898-12

**Authors:** 


Robert C. Turner, a sophomore student of the Medical Department of the University of Buffalo, died at the Buffalo General Hospital, November 18, 1898, aged 21 years. His illness was brief; he was seized three days before his death with a malady that has been nominated Landry's paralysis. He was a promising student, beloved by his associates and teachers. All devoted themselves faithfully to his service in hopes of averting a fatal issue. His remains were taken to his home at Basle, St. Lawrence county, N. Y., for interment.

